# Mechanical Dentistry

**Published:** 1873-07

**Authors:** A. W. French


### ARTICLE II.

### *Mechanical Dentistry.*

BY DR. A. W. FRENCH.

Though the best substitutes for the natural teeth are far from ideal perfection, they, as also those of an inferior character, are of incalculable value to mankind, and reflect as much honor upon our profession as the more conservative arts by which they may be rendered unnecessary. Two generations back of our time, the loss of teeth by disease or accident was an irreparable disaster, (fortunately, we may believe more rare than in our day,) which often drove the subject of it into permanent retirement, years before the march of time had left its unwelcome traces on other features, and the cracked and lisping voice proclaimed to all that misfortune had come, and come to stay; and when, through inscrutable causes, the teeth had one by one all left the hardening and shrivelling jaws, and the power of thorough mastication become only a memory, the chin seeming ever to threaten an assault upon the nose, and the most conspicuous signs of old age sat upon the countenance obvious to all beholders, though perhaps middle life had not yet been reached, the joys of existence, not yet half tasted, were resigned, and hopes of the future were relinquished. Small gaps in the teeth were sometimes filled, for occasions, with wax ; afterwards, feeble attempts were made to secure in the broken ranks the amputated crowns of human teeth, or of the teeth of animals, by the use of wires or silk ligatures, nothing more being expected than the preservation of appearances ; no one anticipating the time when the more essential power of the perfect mouth should be restored. These contrivances were followed by attempts to utilize the semicylindrical and thick enamelled ivory of the tusks of the hippopotamus. Sections of these tusks of the length required for the teeth, were sawn off, and blocks of a sufficient number of teeth were carved in the section, the curved form of the piece enabling the skillful artist to give the block the shape required without cutting entirely through the thick enamel. The base of the blocks was made to assume approximate fit to the jaw by repeated cutting and trying. The blocks were retained in the mouth by wires or ligatures passed through a hole at each end, and tied to any remaining teeth there might be. When there were no teeth to which to secure them, the dentist was not needed. His powers were not equal to the exigency. These attachments were replaced from time to time, by the dentist, and my experience, I may say in passing, goes back to the time when these blocks were worn, and though I never had occasion to make them, I have replaced the ligatures on some of them. It is believed that these structures were among the first attempts to restore masticating teeth; and as we see new sets of teeth which seem to have been made on a model from some other mouth than that in which we see them, become in time not only endurable, but useful, and apparently indispensable to the wearer, so these clumsy masses of ivory, if their supports were not dragged out of the jaw by them, were found, after long use, to become comfortable and even quite useful to the wearer. But when the art of taking impression of the jaws came into use, one half of the difficulties of making artificial teeth were overcome, as a plate of silver or gold could be fitted to the jaw, and the teeth, whether human, or sheep, or carved ivory, were adapted to the plate, and nothing was lacking but teeth composed of some material, which, while it presented a natural appearance, should not absorb the fluids of the mouth, as all the substitutes yet used did, to a degree quite offensive to taste and smell. These were not long wanting, and the germs, if I may so call them, of the present porcelain teeth, were invented. The smaller the number of the teeth required in any case, the surer the operator felt the success in the insertion of plates. A safe and sure fastening was a great desideratum, and whole upper sets were held in place by springs attached to rings or caps placed around lower teeth; and it was many years after it was found that an upper plate which had been long worn, would stay in place without visible attachments, before springs were found to be not indispensable, in cases of both upper and lower sets. The sense of insecurity which most people experience when they first receive a full set of teeth, it was supposed would be permanent unless the plates were in some way secured, and springs were applied to every set. The methods of taking impressions, the soft metals used for models, and the very imperfect swedging of the plates, sufficiently account for the length of time it required to learn that the pressure of the air was a force which could be utilized in the retention of sets of teeth in the mouth. Articulation of teeth, especially in cases of full sets, was imperfectly understood and performed, and this leads me to make a remark or two on that part of the mechanical dentist's work.

An accurate impression is not of more importance in the construction of full dentures than exactness in articulation. This, indeed, may make all the difference between success and failure, and will make as wide a difference in the aspect of the patient as in the ability to properly and easily masticate the food. The adhesion of the plate to the jaw may be very slight, and yet the teeth perform their functions without getting out of place, if the grinding surfaces come together correctly, while the plates would need to be riveted to the jaws to keep them immovable, where the articulation was very imperfect. Good articulation is not gained by the antagonism of the faces of the teeth only, but it is requisite that the space between the jaws should be properly divided between the two arches, so that neither shall be too long or too short. For determining this, I know no rule. The good judment of an experienced dentist must decide for each case, and an error here may be attended by unpleasant consequences, though the fault may be unsuspected. An excess of length in the lower set is not quite as fatal as the reverse, but it will cause the patient much and protracted embarrassment.

When we are required to adapt a lower set of teeth to an upper set of natural ones, which from having had no antagonists for a considerable length of time, have come down from their places two or three lines, though still firm in their sockets, the division of space is not left to us. It is with difficulty that a set can be made entirely satisfactory under these circumstances. If, however, the grinding surfaces are accurately adapted to one another, the wearer may overcome all difficulties, and the teeth become useful in time. A contrivance of nature, by which the masticating power is greatly increased, the arched form of the grinding faces of the molars and bicuspids, which may be observed in complete sets of natural teeth, is not often imitated in the sets most commonly used at the present time. This effect could be best produced in the construction of the continuous gum teeth; next with plain plate teeth, and gum plate teeth; but is hardly attempted with the blocks now so generally employed. A strait line from the canine to the last molar, defines the shape of a large proportion of all the sets we now see. This error in construction renders the teeth much less useful and effective than they might be, but it simplifies the dentist's work, and helps him to cheapen his product and adapt it to the demands of his market. This error is accompanied by another which is sometimes made necessary by it. This is the even occlusion of the incisors, or the "closing square," as it is sometimes called; a faultry plate may be made practicable or wearable by turning in the superior incisors so that they shall meet the lower ones, and the foward tilting be prevented. But this misarrangement is at the expense of all naturalness and expression, and clearness of vocal articulation. It, however, reduces the labor and the study of the operator to a minimum, and is, accordingly, extremely common. Perhaps a major part of the whole sets of teeth now worn, both upper and lower, presents a perfect plane on the griding and cutting surfaces, and if turned down on a table every tooth would rest on the board. This we all know is a great departure from our model, nature; but long use of these imperfect substitutes for natural teeth makes them tolerable to the wearer, and bearable to the viewer. They will continue in general use until the people, being more enlightened, call for a higher grade of culture in the dentist, and the display of more art in his work.

It is believed that nothing has occurred since the last meeting of the Illinois Association to change the views of any member on the resolution then unanimously passed, that the good of both dentists and patrons would be promoted by a general abandonment of rubber, and a return to the use of metal plates. I have observed with care the effects of wearing rubber, next to the mucous membrane in all the cases that have presented; and in nearly all there was a turgidity of the vessels of the membrane indicative of irritation or of heat, which is not seen where metal plates are worn. Chronic nasal catarrh has made its appearance in several persons coincident with the wearing of rubber plates, and further investigation may demonstrate that the one was the cause of the other. It may be well for all dentists to scrutinize closely the condition of all mouths in which rubber is worn, and we can learn in time to what extent it is deleterious. Without pretending to give satisfactory reason for the fact it is a matter of observation that absorption goes on much more rapidly under a rubber than a metal plate, and the adhesion is consequently sooner lost. This fact, making it necessary to renew the teeth quite often, may be a sufficient argument with economical people to persuade them to choose gold as being equally cheap when the expense is spread over a long period of time. Absorption, however, in some cases, is constantly and steadily taking place, whatever material has been chosen for the plates, and in time the ridge of both jaws entirely disappears, until the surface to be covered by a plate is as flat as the palm of the open hand.

Fortunately in most of such cases the soft tissues have not been absorbed to the same extent as the bone, but have shrivelled or gathered into the smaller space left for them, and from a soft cushion for the plate, to which it readily adapts itself, and affords sufficient adhesion to retain the plate in place. When this is not so, and the palate is at once flat and hard, the leverage of the teeth, owing to their extreme length, being considerable, the best fitting set of teeth may not be entirely satisfactory without extraneous supports.

In the construction of *partial* sets, the opinion that gold should be exclusively used has been so often and so generally expressed by all dentists, that it is not my purpose to urge that practice here. It looks a little like imposing upon a good natured patient to cover his entire palate with rubber two lines in thickness, in order that a vacancy or two in his incisors may be filled. It would appear to be the substitution of a greater for a less evil. A very small plate of gold will sustain and keep in place several teeth, if adapted in the best way. In some very deep jaws, of which you get but imperfect impressions, and where a broad plate will be a little less perfect in its adaptation than the impression, a quite narrow plate just resting on the ridge to which it may be easily fitted, may be secured by clasps embracing either bicuspids or molars. If teeth are short, and no space exists in which a clasp may be inserted without fileing, a pair of little horns on each end of the plate, may be made to embrace simply the necks of the teeth chosen to support the plate. These hooks can be sprung into their places and will give sufficient support to the set. As the embracing part is but little thicker than the plate, it need cause no more decay than a close fitting suction plate. Our aim should be to place in the mouth as little matter as will answer our purpose, and we can often make a comfortable and durable set, either of a few or many teeth, which shall have less bulk and occupy less space than did the natural organs, with their gums, alveoli, &c. In choosing between clasps and atmospheric pressure, I am sometimes influenced by the degree of refinement of the patient—having learned that very coarse and rough persons, for whom hooks of steel would be none too strong, will soon destroy the delicate adaptation necessary for the latter, while persons of a different nature may wear them till the natural teeth by their side have all departed.

For incisors, I am in the habit of using plain teeth, unless there has been such an amount of absorption as to give plenty of room for artificial gums, regarding it a common mistake to attempt to force in gums where the space is already occupied. If the elevating of the lip displays the plate, the latter can be cut away around the teeth. Plain teeth have the advantage that they can be put on with proper and natural inclinations, (which is far from the case with gum teeth,) and a correct expression can be given to them. The margin of the plate should be made very thin, there being just sufficient gold to cover the base of the tooth.

Our first and greatest efforts should be made to preserve natural organs; but when through our inefficiency or the neglect of the patient, these are gone past recovery, the skill of the mechanical dentist can do much to restore youthfulness and health, and place the patient back to that period of life when the ravages of caries were unknown to him. Indeed, many people who had suffered almost constantly, through childhood and youth, all the pains attending the gradual dissolution of the teeth, look back with no regret to their total loss, and deem the day which witnessed the removal of the last of them, a day of happy deliverance from intolerable suffering. This joy would not be unmixed with grief, if the disfigurement and incapacity caused by their loss was to be permanent and life long. But the mechanical dentist has restored their features and their powers without renewing their sufferings; and he may be ranked among the benefactors of his race. His services are gratefully remembered by those who enjoy the results of them. One lady who had worn sets of teeth for many years, witnessing the writhing of her daughter under the operation of preparing a cavity to fill, exclaimed, with the deepest feeling in the tone of her voice, "I *pity* those people who have natural teeth." A gentleman said to me that his wife would not part with her set of artificial teeth for all the natural ones in the world. Another said that he would not exchange his artificial set for any natural ones he ever had. Many others speak in tones of profound gratitude of the comfort bestowed by the hand of the painstaking dental artist. But these expressions are doubtless familiar to you all, and should and do serve as a lively stimulus to your resolves to do the most and best you may for the amelioration of the sufferings of mankind.

---

